# Supplementary material for: Design, Synthesis and Cytotoxic Activity Evaluation of Newly Synthesized Amides-Based TMP Moiety as Potential Anticancer Agents over HepG2 Cells
Source: Molecules. 2022 Jun 20;27(12):3960. doi: 10.3390/molecules27123960 (PMC9227250; doi:10.3390/molecules27123960)
Supplement: Supplementary file 1 [file molecules-27-03960-s001.zip › molecules-1730734-supplementary.pdf]

Supporting Information

Design, Synthesis and Cytotoxic Activity Evaluation of Newly Synthesized Amides-based TMP moiety as potential anticancer agents over HepG2 cells

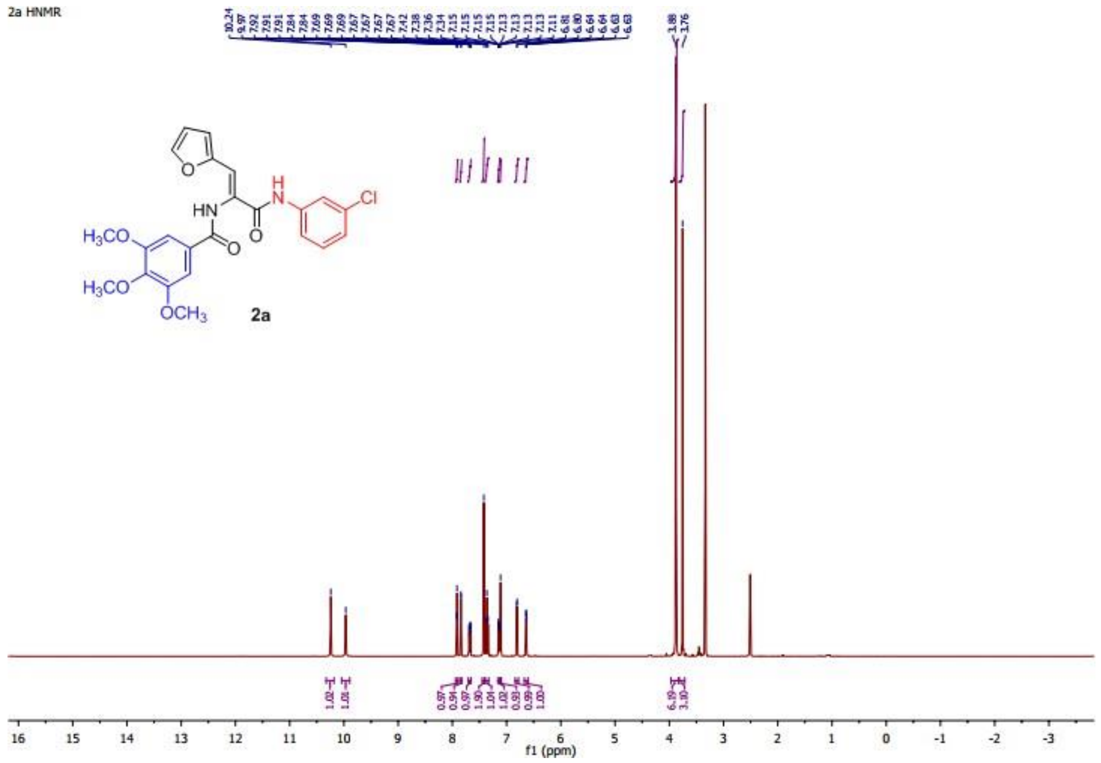

Figure S1: ^{1}H-NMR spectrum of compound **2a**

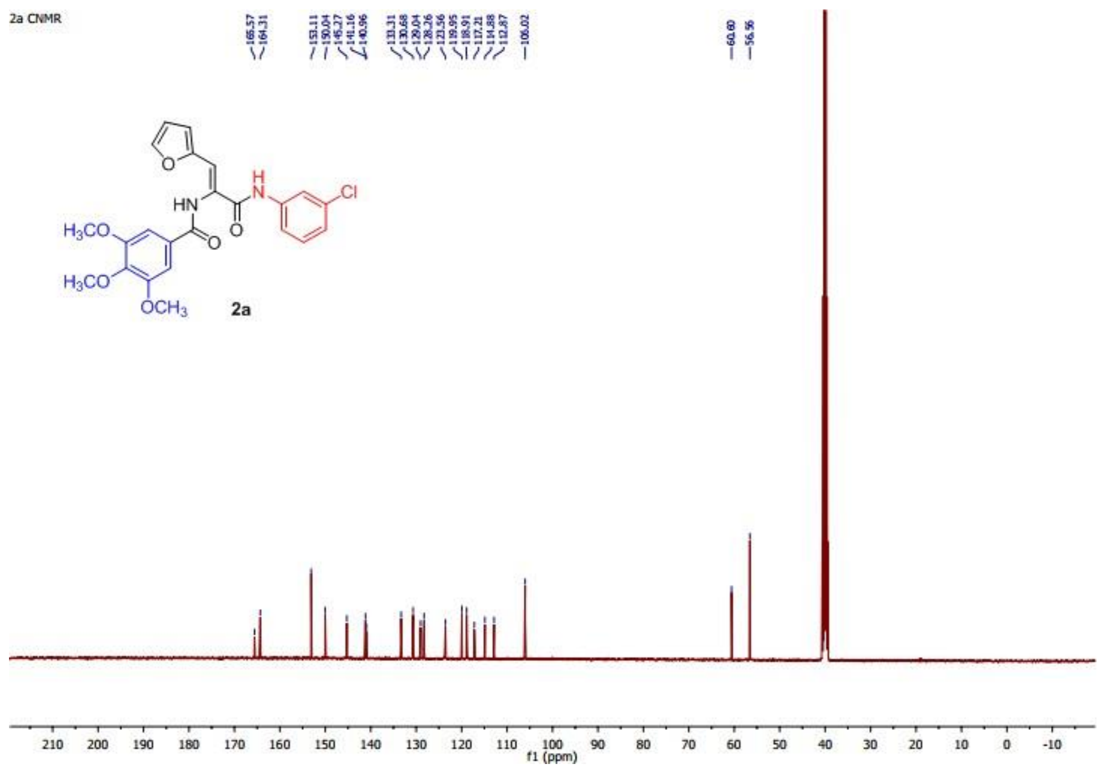

Figure S2: ^{13}C-NMR spectrum of compound **2a**

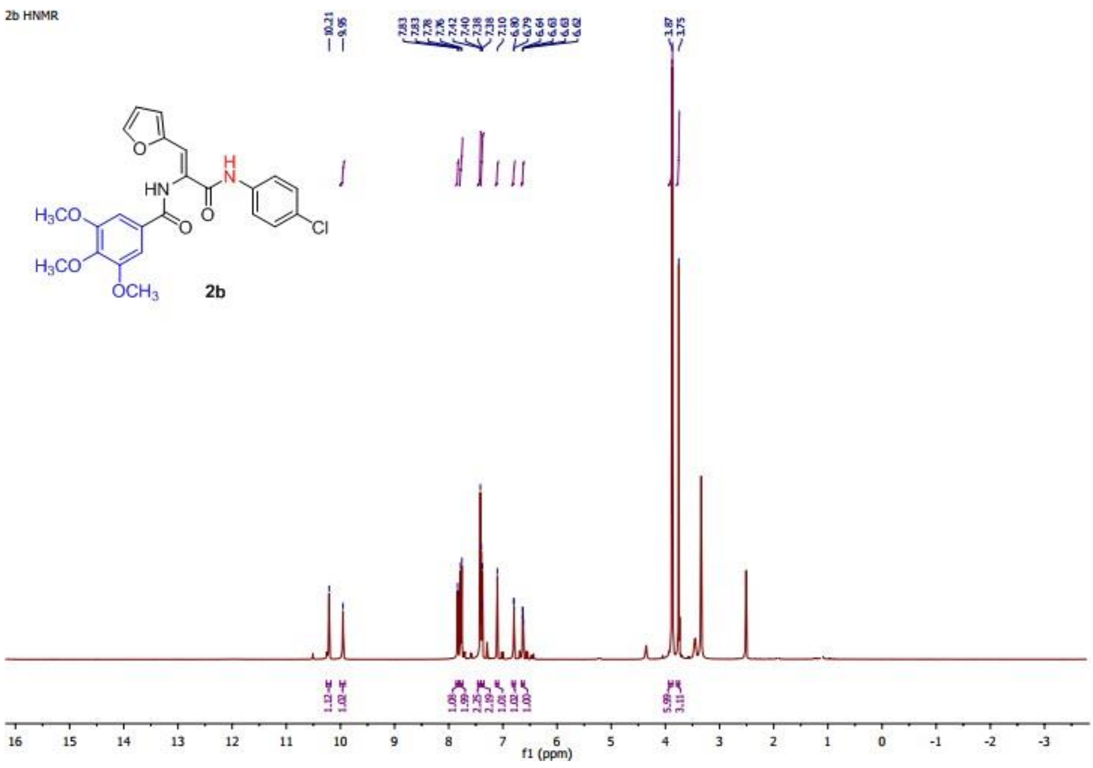

Figure S3: ¹H-NMR spectrum of compound **2b**

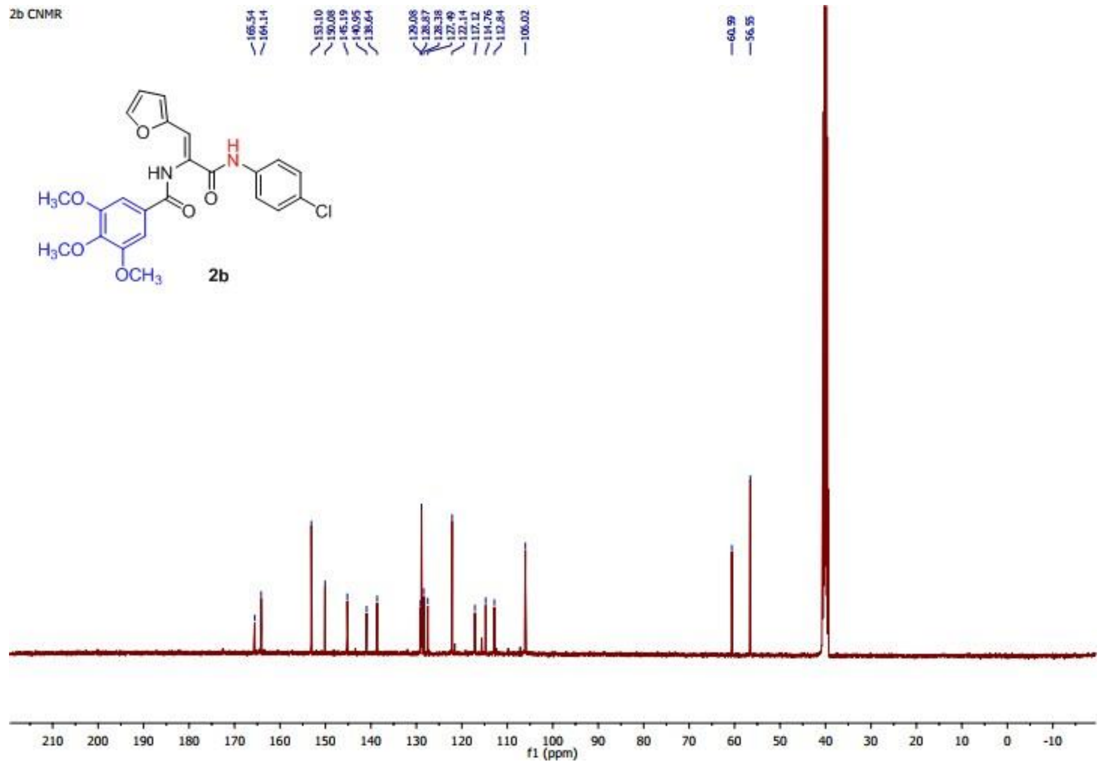

Figure S4: ^{13}C-NMR spectrum of compound **2b**

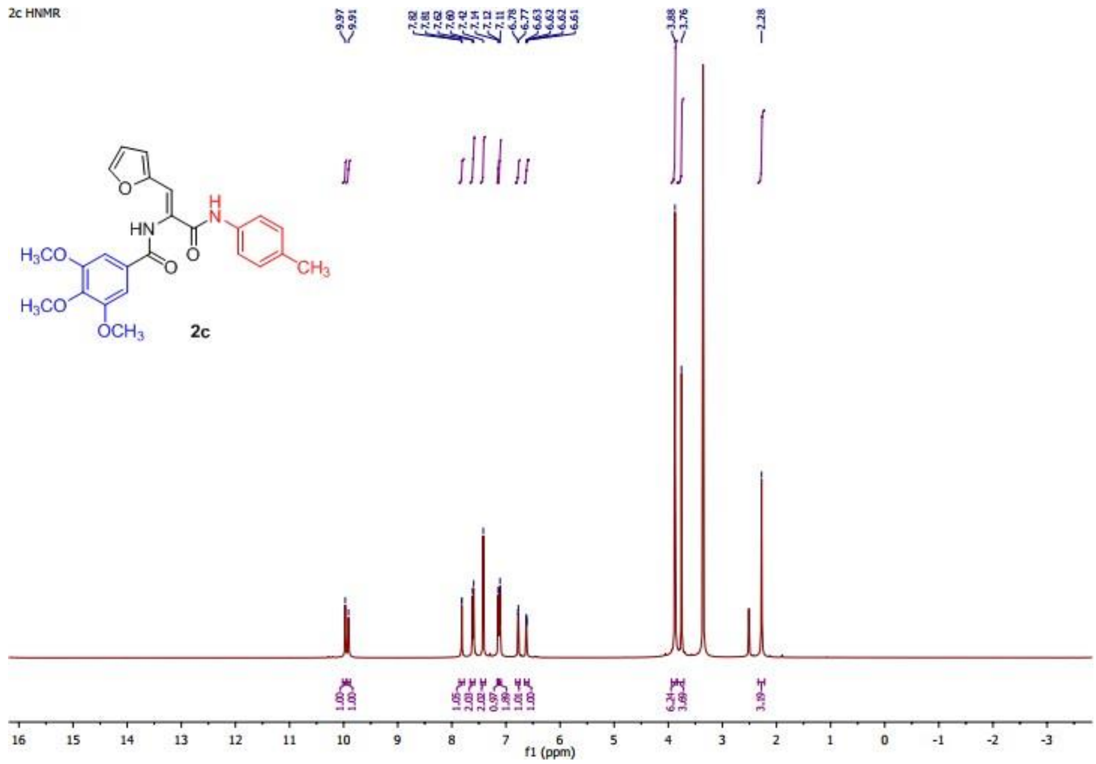

Figure S5: ^{1}H-NMR spectrum of compound **2c**

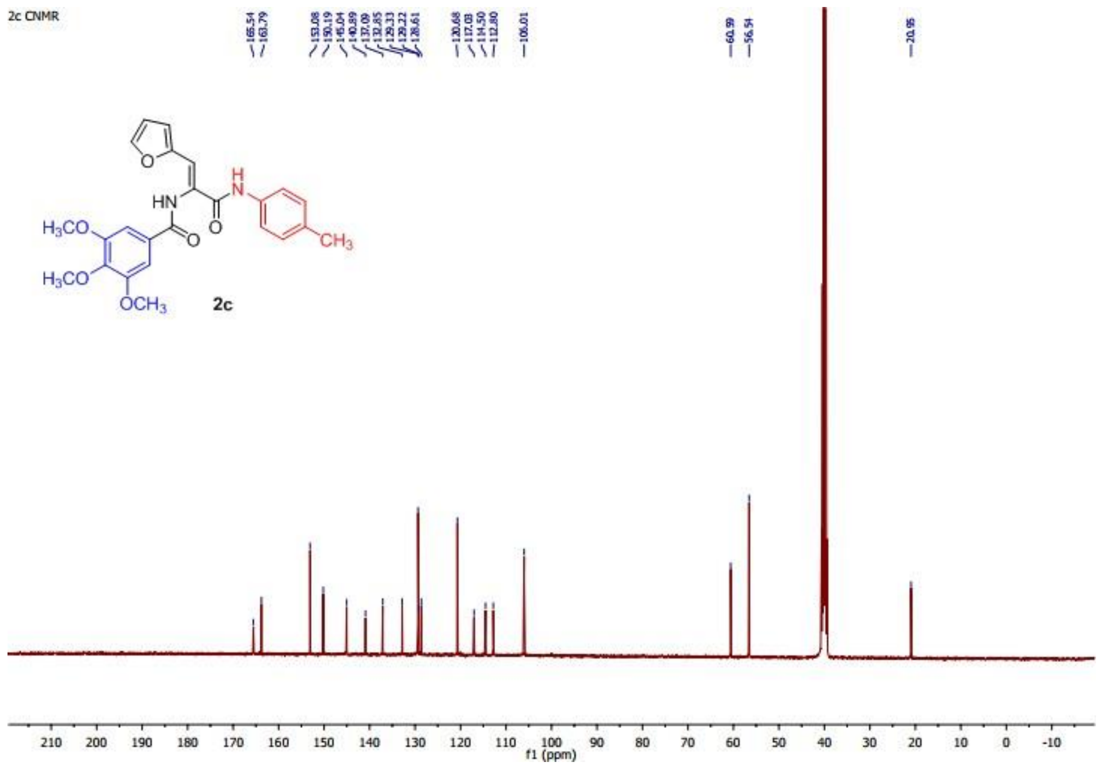

Figure S6: ^{13}C-NMR spectrum of compound **2c**

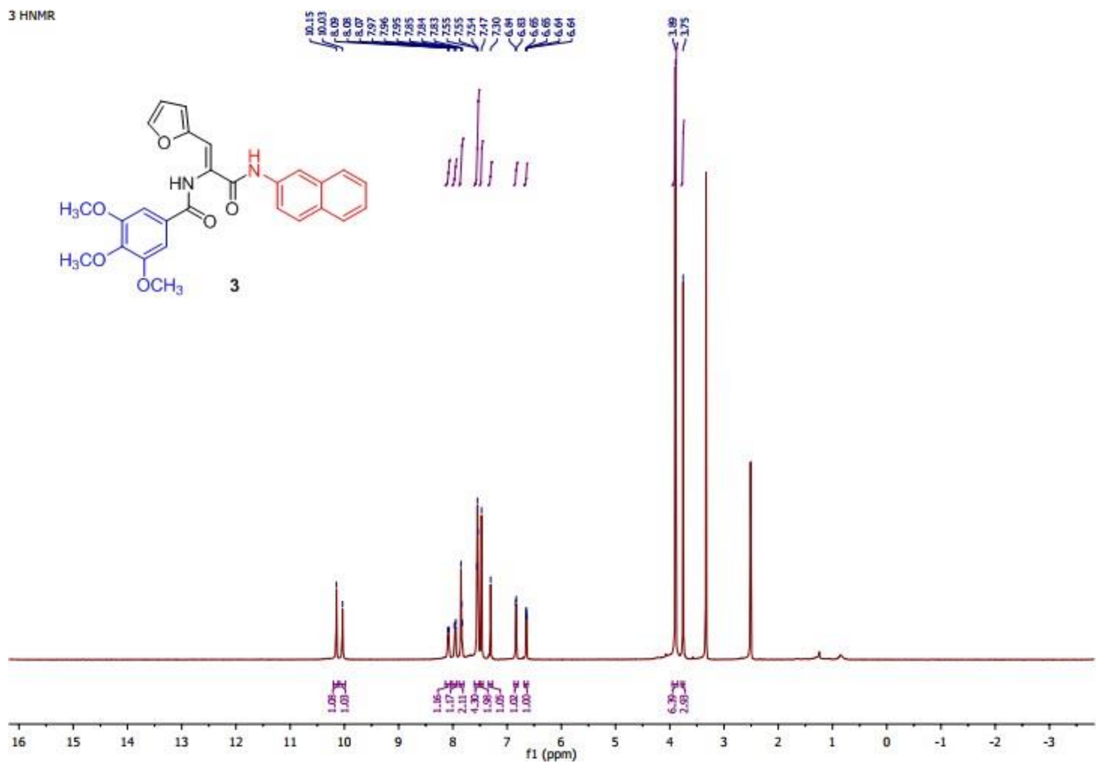

Figure S7: ^{1}H-NMR spectrum of compound **3**

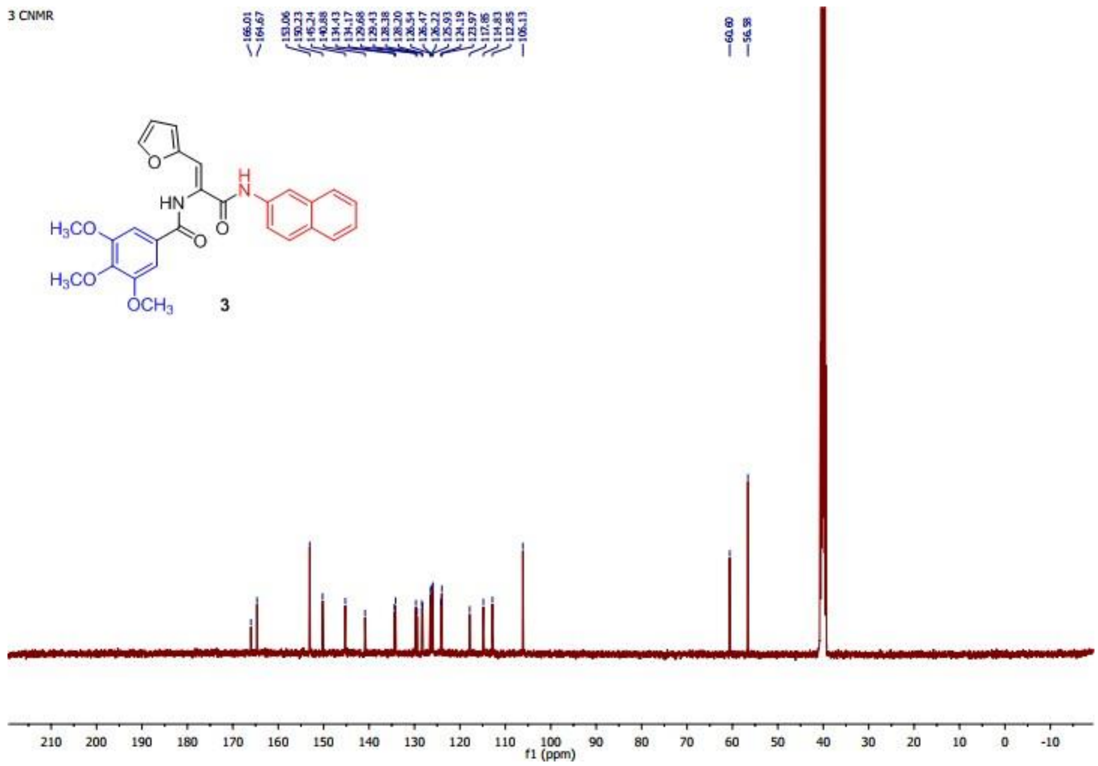

Figure S8: ^{13}C-NMR spectrum of compound **3**

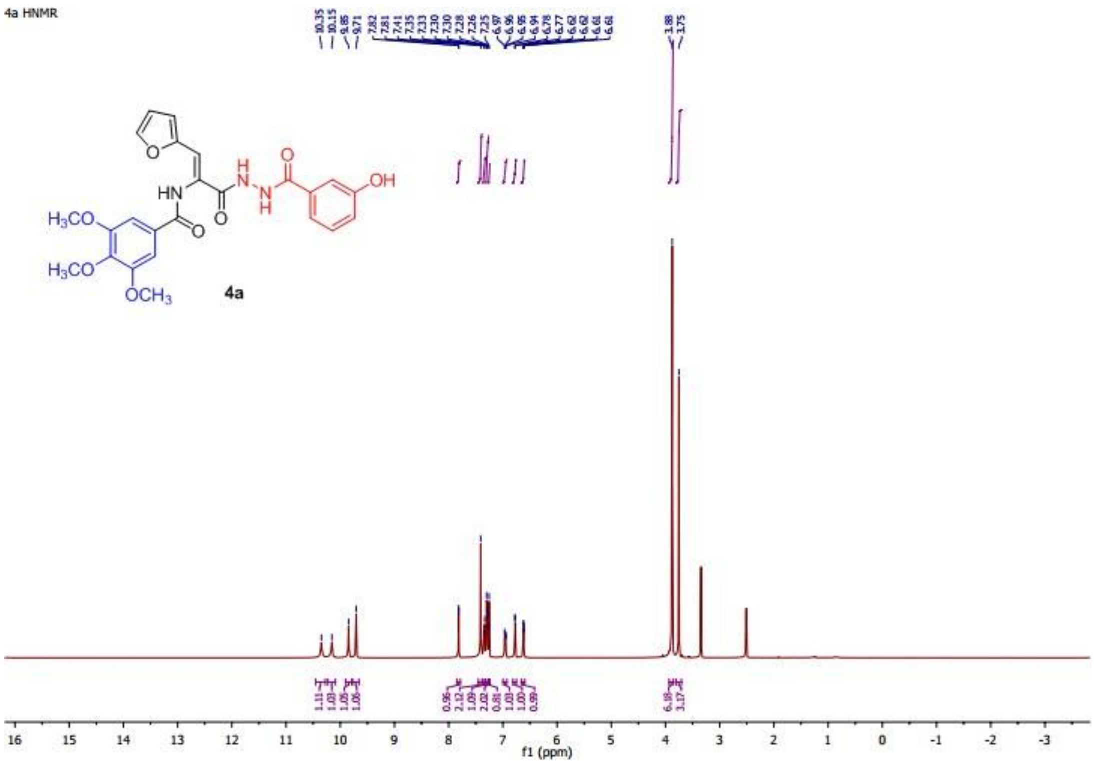

Figure S9: ^{1}H-NMR spectrum of compound **4a**

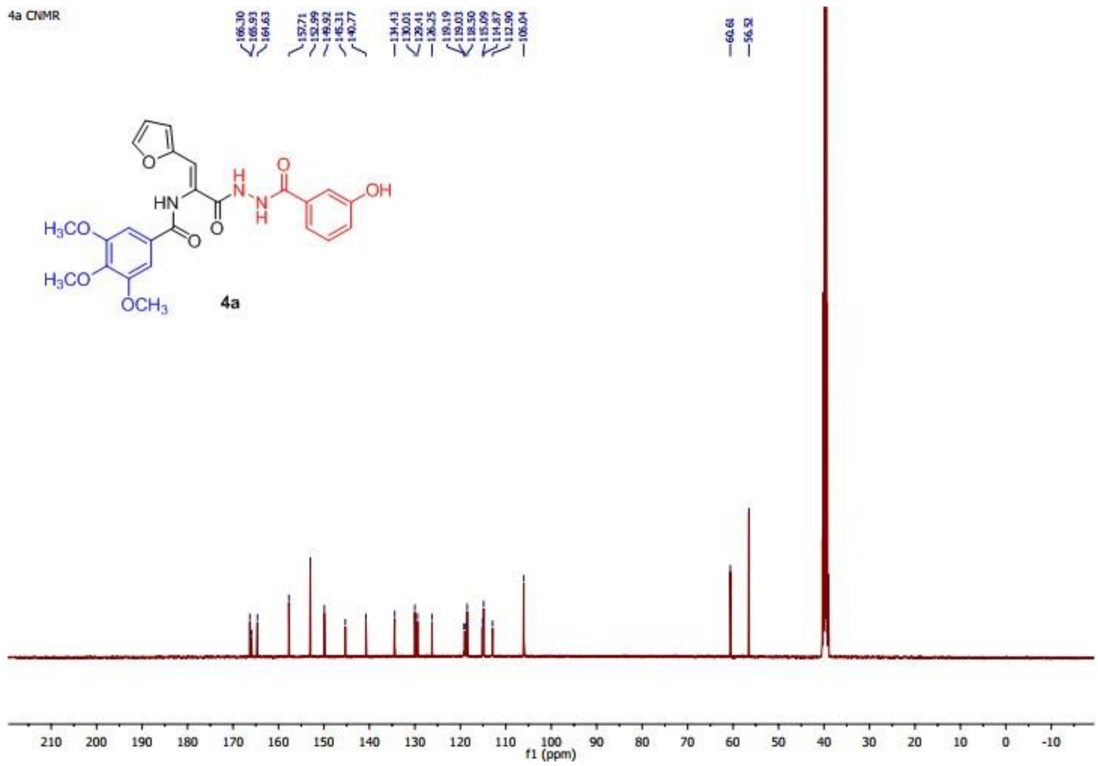

Figure S10: ^{13}C-NMR spectrum of compound **4a**

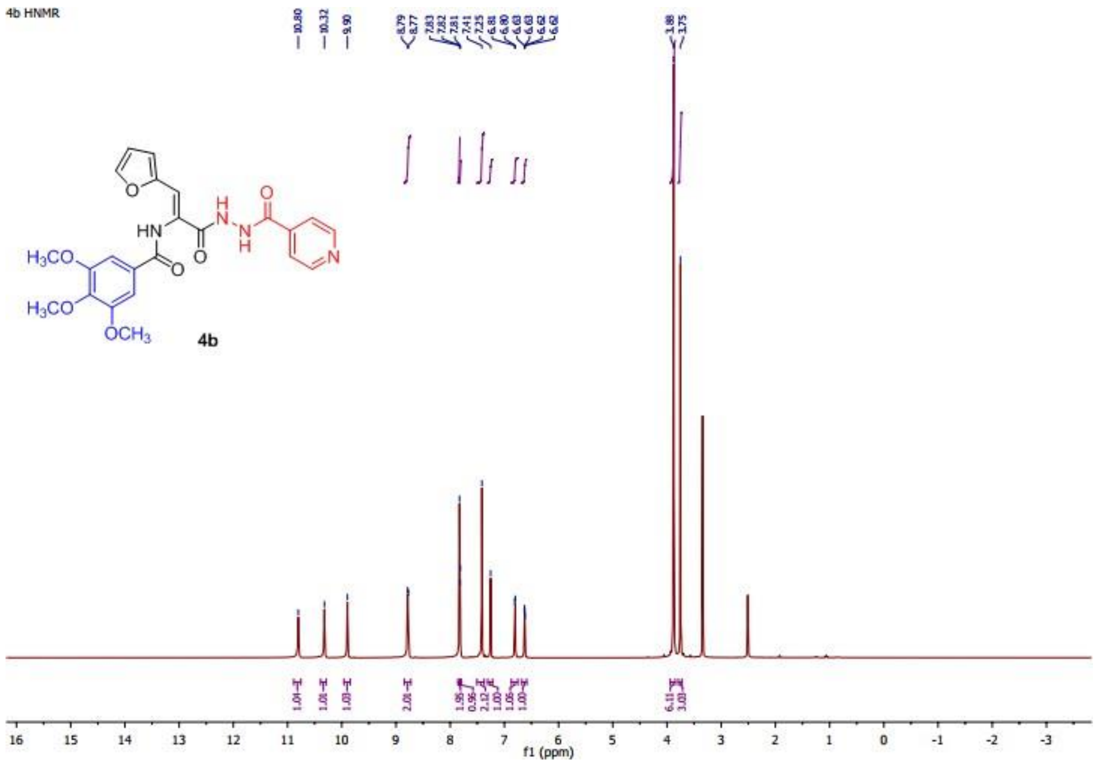

Figure S11: ^{1}H-NMR spectrum of compound **4b**

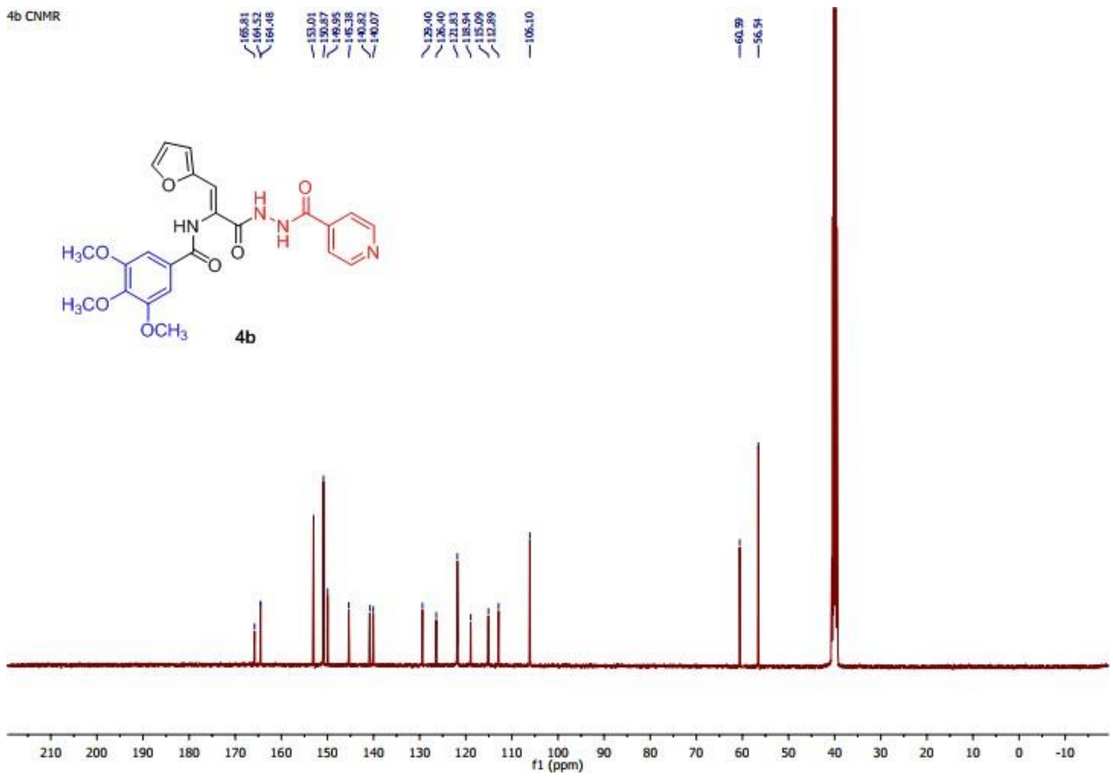

Figure S12: ^{13}C-NMR spectrum of compound **4b**

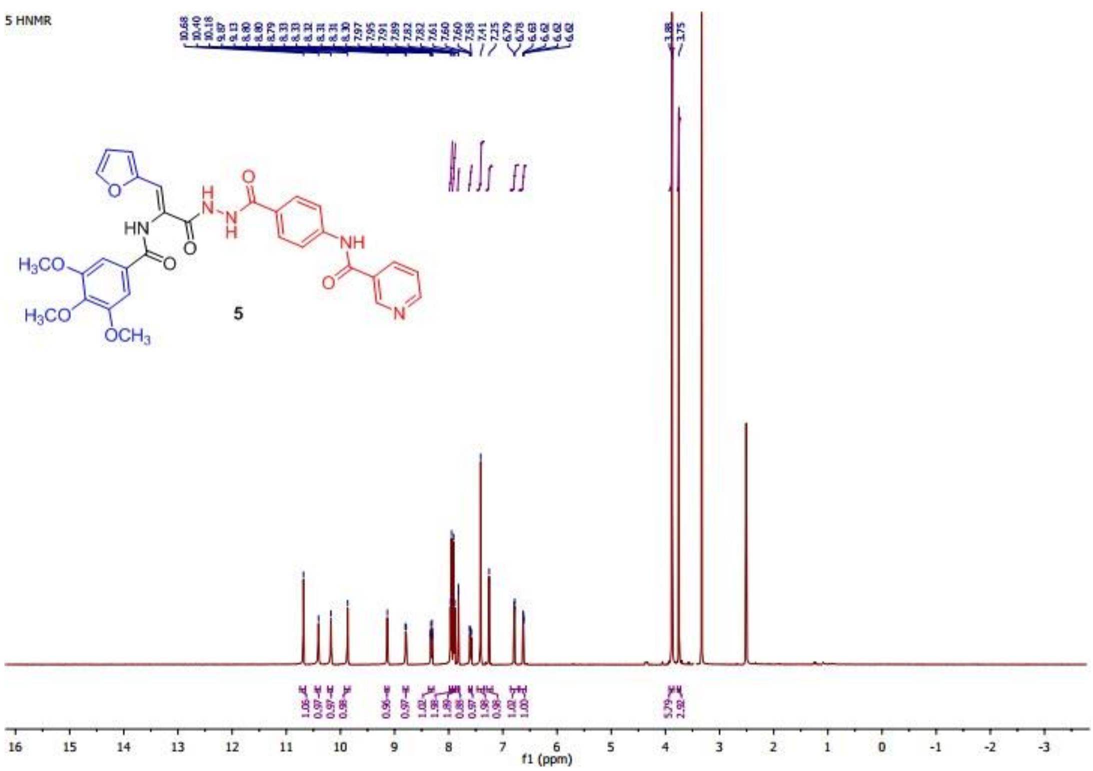

Figure S13: ^{1}H-NMR spectrum of compound **5**

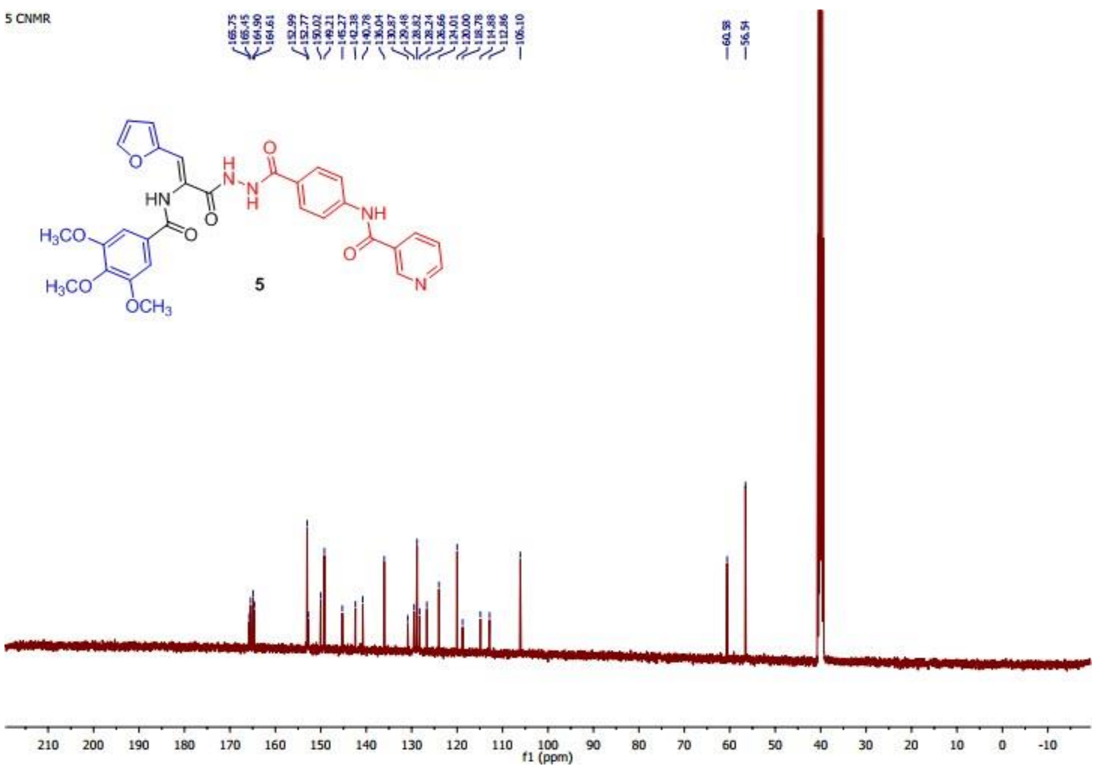

Figure S14: ^{13}C-NMR spectrum of compound **5**

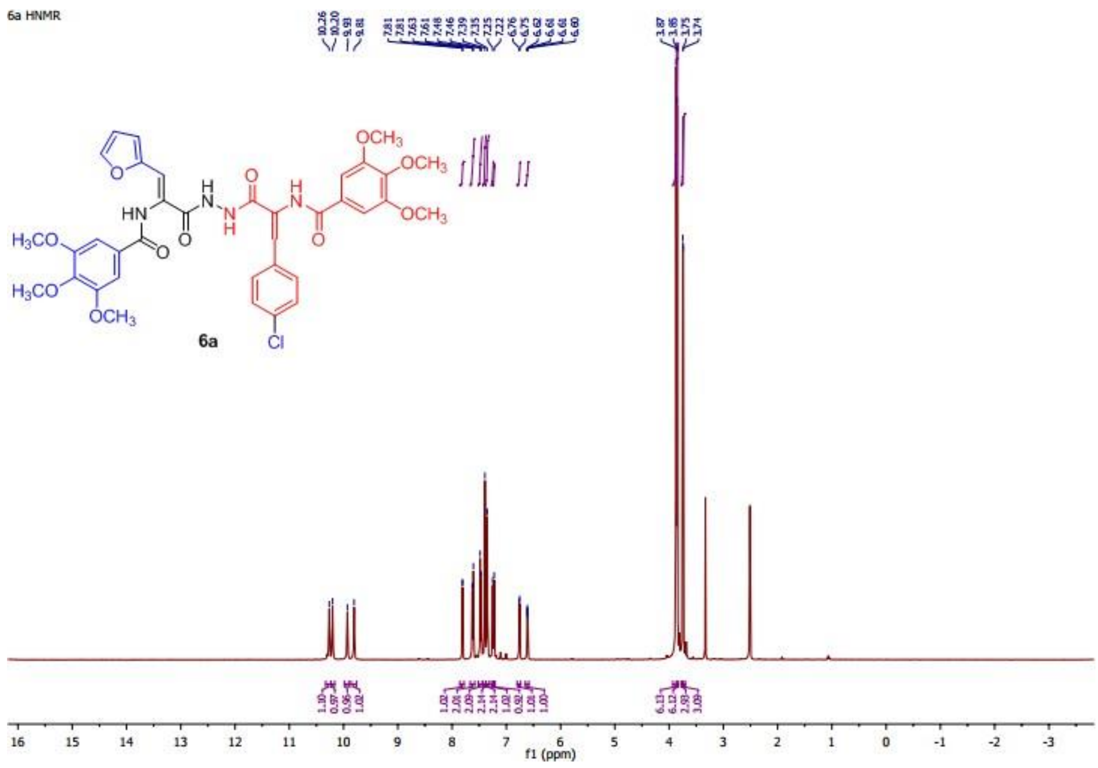

Figure S15: ^{1}H-NMR spectrum of compound **6a**

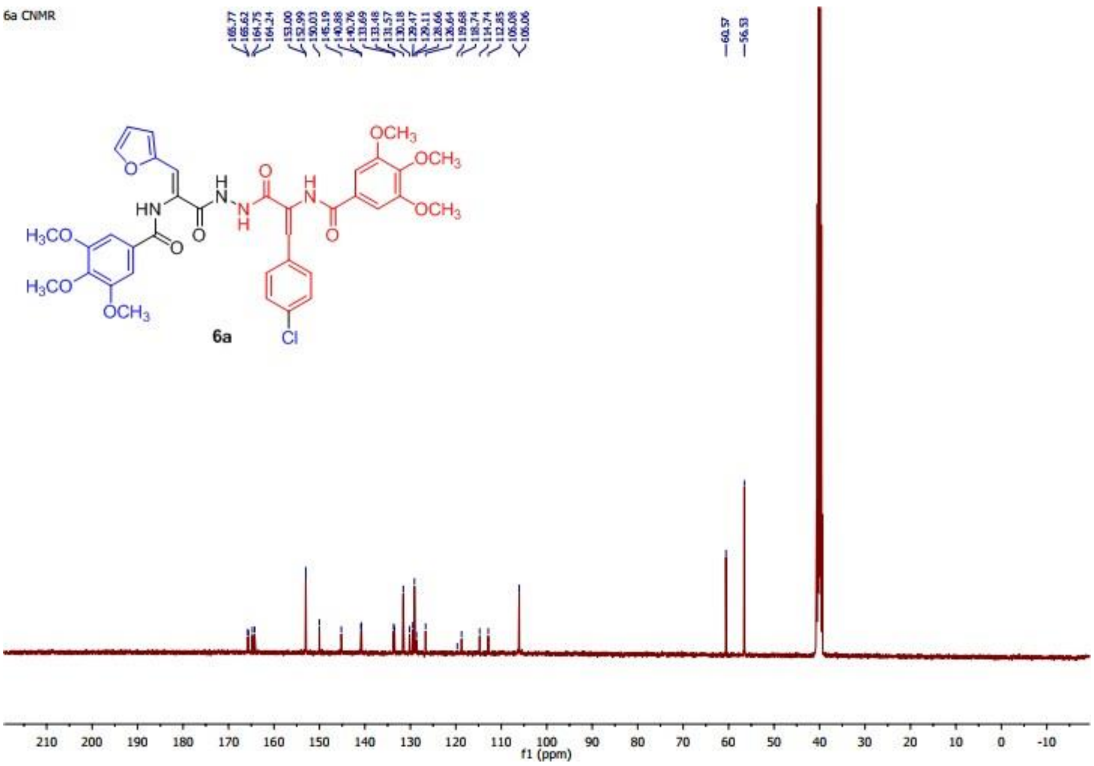

Figure S16: ^{13}C-NMR spectrum of compound **6a**

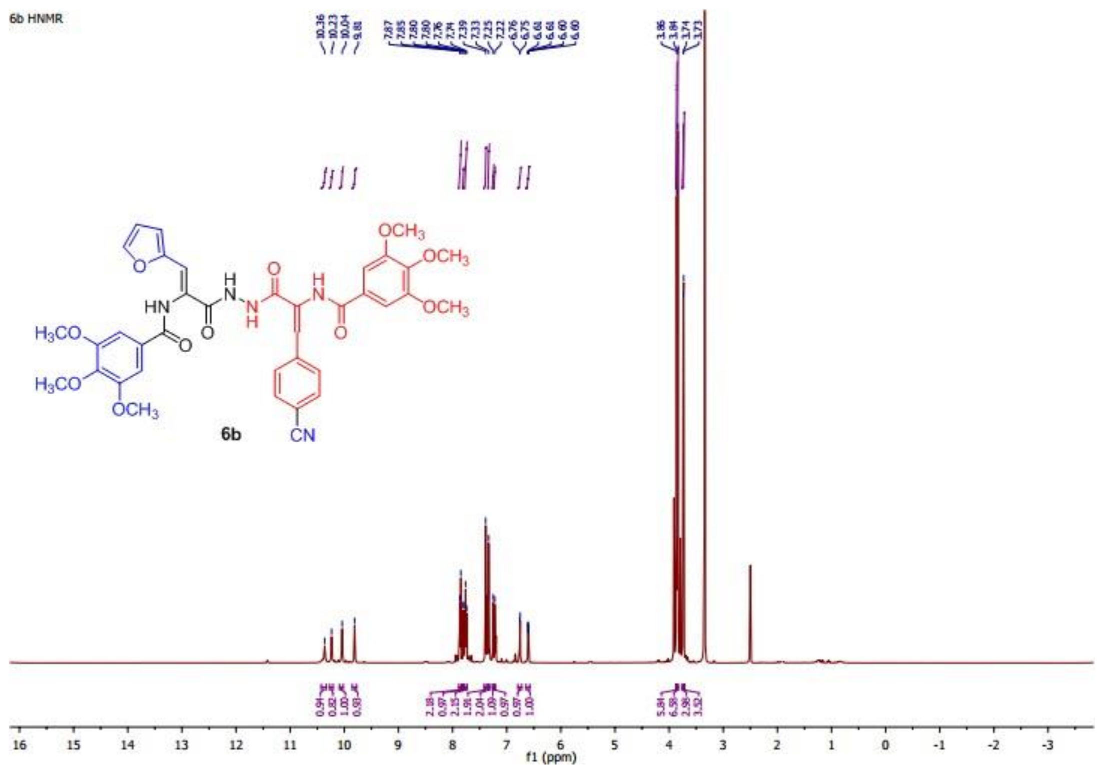

Figure S17: ^{1}H-NMR spectrum of compound **6b**

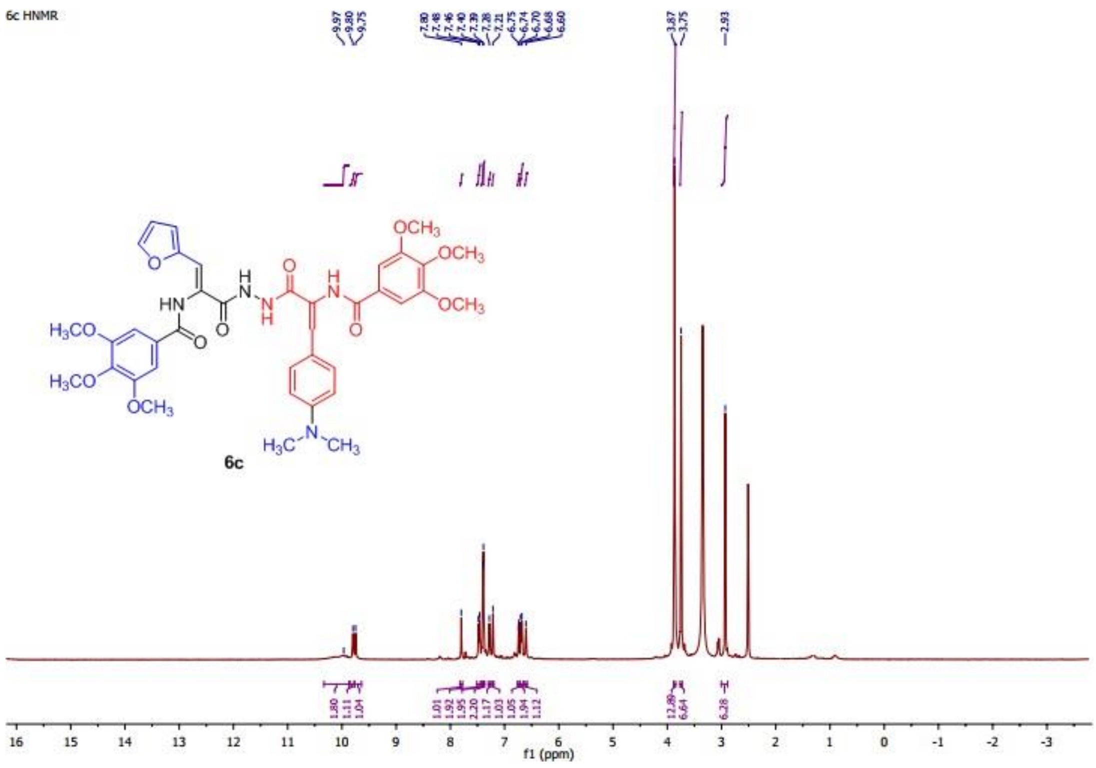

Figure S18: ^{1}H-NMR spectrum of compound **6c**

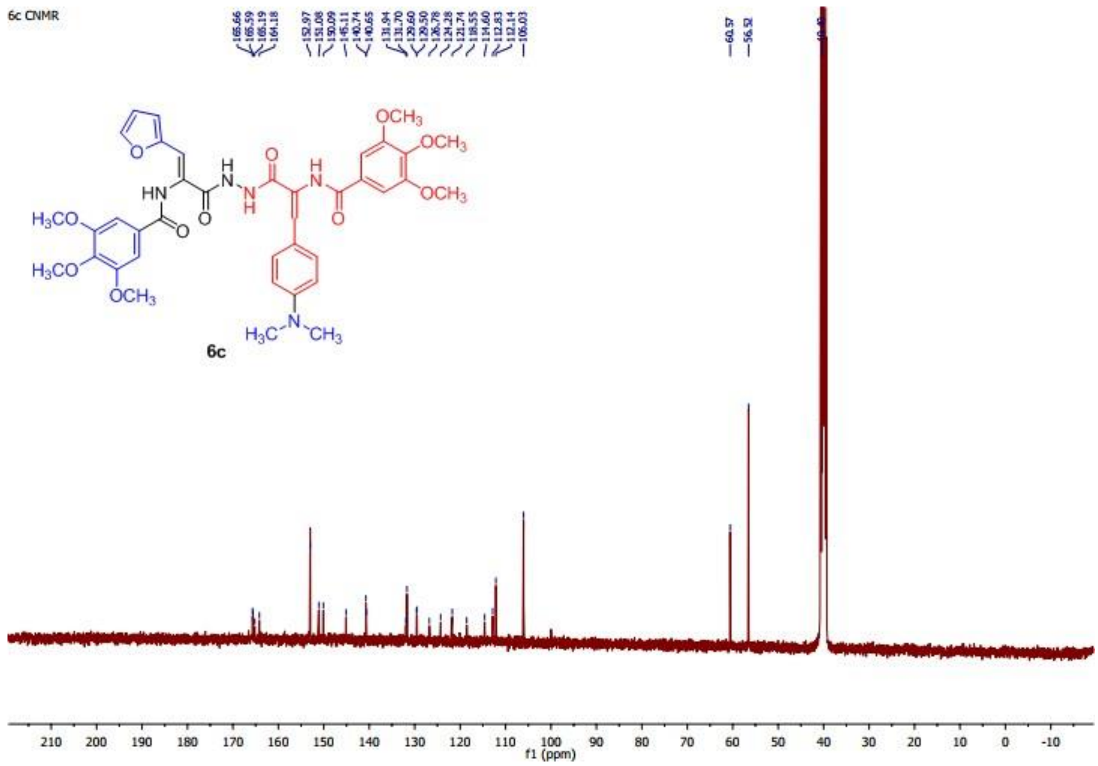

Figure S19: ^{13}C-NMR spectrum of compound **6c**

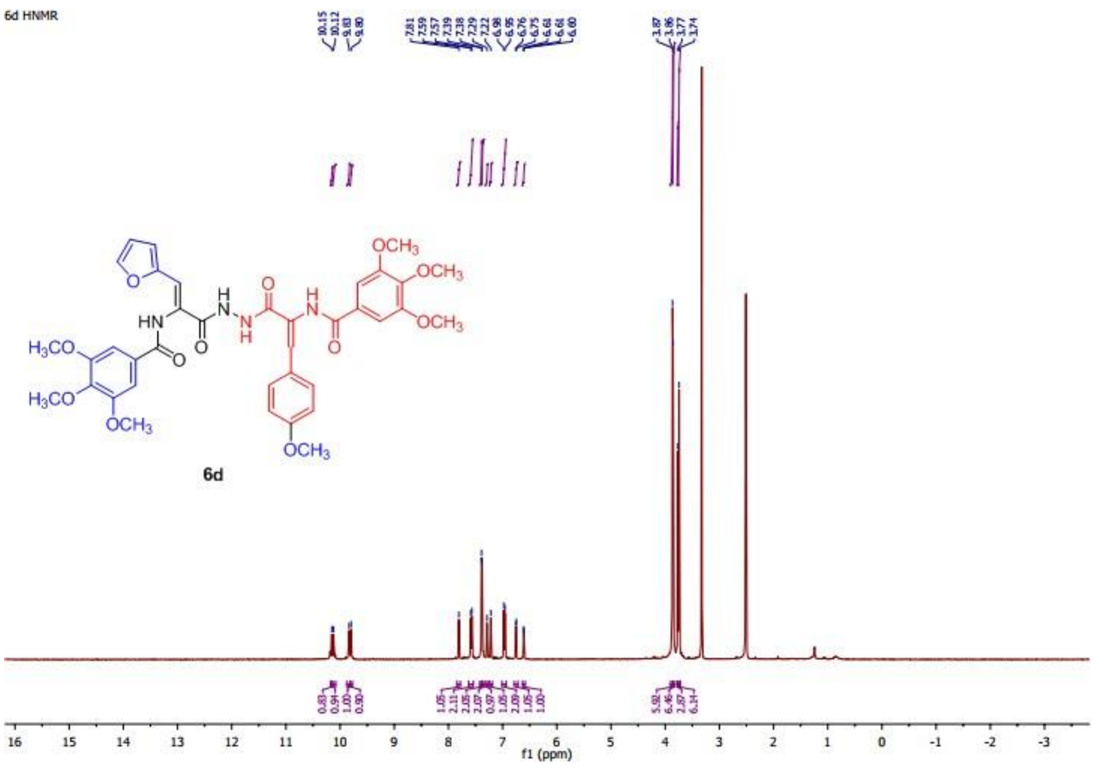

Figure S20: ^{1}H-NMR spectrum of compound **6d**

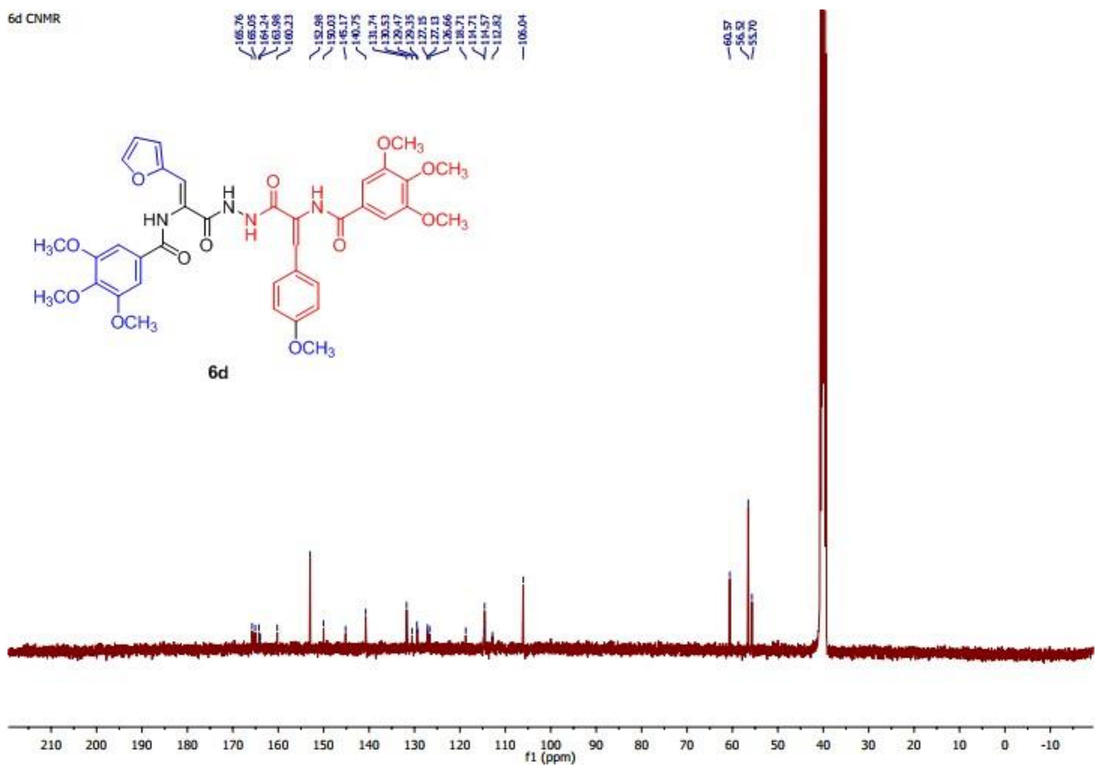

Figure S21: ^{13}C-NMR spectrum of compound **6d**

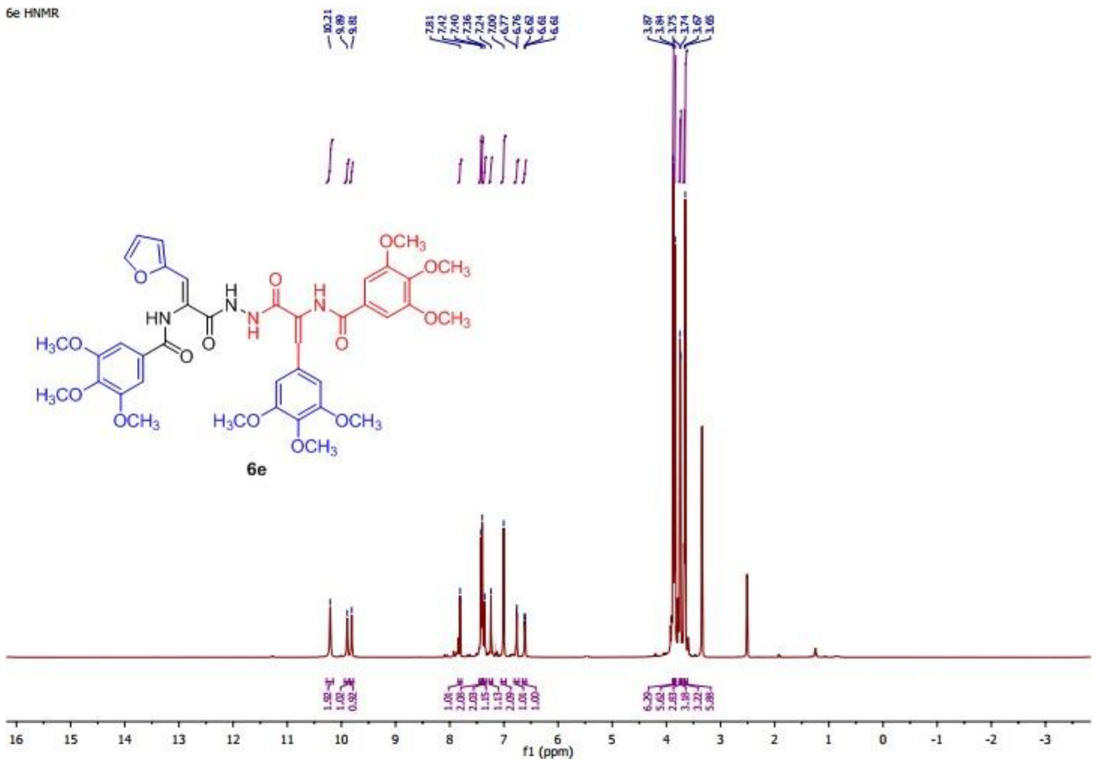

Figure S22: ^{1}H-NMR spectrum of compound **6e**

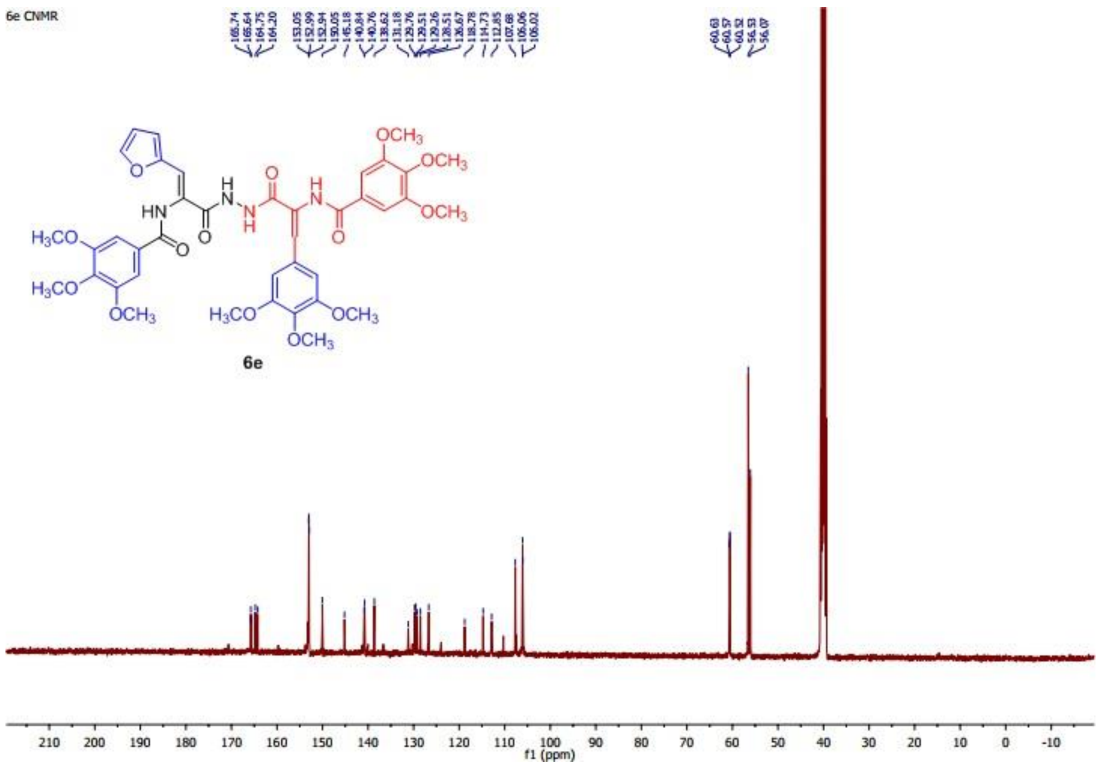

Figure S23: ^{13}C-NMR spectrum of compound **6e**

4.1. Chemistry: General

Melting points were determined in open capillaries tube using Electrothermal Digital melting point apparatus and were uncorrected. ^{1}H-NMR and ^{13}C-NMR spectra were obtained with a Bruker 400 MHz DRX-Avance NMR spectrometer, peaks positions are given in ppm downfield from tetramethylsilane (TMS) as the internal standard. Elemental analyses were performed on Elementar, Vario El, Microanalytical unit, Cairo, Egypt and were found within ±0.4% of the theoretical values. Chemicals and reagents were obtained from commercial sources and used without further purification.

4.3. Biological studies

4.3.1. Cytotoxic activity evaluation

To measure the cytotoxic actvity of the amide derivatives **2-6e** in hepatocellular carcinoma (HepG2) cell line (ATCC Cat No. HB-8065) as well as normal human liver cell line HL-7702 (ATCC Cat. No. 77402), cell viability assay was assessed using MTT assay method. Cells at density of 1 x 10^4 were seeded in a 96-well plate at 37 ^oC for 24 h under 5% CO_2. After incubation, the cells were treated with different concentrations of the test schiff bases **2-6e** and incubated for 24 h, then 20 μl of MTT solution at 5 mg/mL was applied and incubated for 4 h at 37 °C . Dimethyl sulphoxide (DMSO) in volume of 100 μl was added to each well to dissolve the purple formazan that had formed. The color intensity of the formazan product, which represents the growth condition of the cells, is quantified by using an ELISA plate reader (EXL 800, USA) at 450 nm absorbance. The experimental conditions were carried out with at least three replicates, and the experiments were repeated at least three times.

4.3.2. *In vitro* HDAC inhibition assay

The *in vitro* HDAC inhibitory activities of compound **6a** and SAHA against two HDAC isoforms (HDAC1, 2) were measured using ELISA assay kits {Mybiosource, Inc. [#MBS2020012 and #MBS2510971]} according to manufacturer's directions. Briefly, HepG2 cells were trypsinized, counted and seeded at density of 2 x 105 into 96-well micro titer plates. Cells then were incubated in a humidified atmosphere at 37 ^oC for 24 h. The standards, the tested compounds, and the positive reference SAHA were diluted to designated concentrations. On the 96-well micro titer plates standard or sample was added to each well in 100 μL, and incubated at 37 ^oC for 2 h. The solution was aspirated and 100 μL of prepared. Detection Reagent A was added to each well. Incubation was done at 37 ^oC for 2 h. After washing 100 μL of prepared Detection Reagent B was added and incubation was continued at 37 ^oC for 30 min. Five washings were done, then 90 μL of 3,3',5,5'-tetramethylbenzidine (TMB) substrate solution was added and incubated at 37 ^oC for 15-25 min. Stop solution was added in 50 μL. Cells were exposed to different concentrations of compound **6a** and SAHA (0.01, 0.1, 1, 10 μM) for 72 h. Optical density (O.D.) was measured at 570 nm using microplate reader (Spectromax Plus 96 well plate spectrophotometer), and the

concentration that induces 50% of maximum inhibition of HDAC isoforms (IC_{50}) were determined.

4.3.3. Tubulin inhibitions Assay

Compound **6a** was evaluated for its tubulin inhibitory activity according to manufacturer's instructions using # abcam Human Beta-tubulin simplestep ELISA Kit ab245722.

4.3.4. Cell cycle analysis of compound 6a

Cell cycle analysis in HepG2 cells was investigated using fluorescent Annexin V-FITC/ PI detection kit (*BioVision* EZCellTM Cell Cycle Analysis Kit Catalog #K920) by flow cytometry assay. HepG2 cells at a density of 2 × 105 per well were treated with compound 6a at its IC50 concentration for 48 h. After treatment, cells were washed twice in PBS, fixed in 70% ethanol, rinsed again with PBS. Cells were collected by centrifugation at 2000 rpm for 5 min and stained with DNA fluorochrome PI for 15 min at 37 °C in dark. The samples were immediately analyzed using Facs Calibur flow cytometer (Becton and Dickinson, Heidelberg, Germany).

4.3.5. Apoptosis assay for compound 6a

Apoptosis in HepG2 cells was investigated using fluorescent Annexin V-FITC/ PI detection kit (*BioVision* Annexin V-FITC Apoptosis Detection Kit, Catalog #: K101) by flow cytometry assay. HepG2 cells at a density of 2×10^5 per well were treated with compound **6a** at its IC_{50} (μM) for 48 h, then the cells were harvested and stained with Annexin V-FITC/ PI dye for 15 min in the dark at 37 °C . The samples were immediately analyzed using *FACS Calibur* flow cytometer (Becton and Dickinson, Heidelberg, Germany).

4.3.6. *C*aspase 3/7 assay for compound 6a

Caspase 3/7 in HepG2 cells was investigated using CellEvent® Caspase 3/7 Green Detection Flow Cytometry Assay Kit. *C*aspase 3/7 activity in HepG2 cell line was detected in the presence of compounds **6a** at its IC_{50} concentration (μM) using CellEvent® *c*aspase 3/7 green detection flow cytometry assay kit according to manufacturer's directions. HepG2 cells at a density of 2.5×10^5 per well were harvested and washed with ice cold PBS, and cell lysates were prepared and

combined with reaction buffer and incubated with specific colorimetric substrates (*C*aspase 3/7 Detection Reagent) at 37 ^{o}C for 6 h. Detection reagent is composed of DNA binding dye conjugated to *c*aspase 3 and *c*aspase 7 recognition sequence (DEVD) by a short peptide linker. The released DNA binding dye binds DNA producing a bright and stable signal. Finally, the samples were analyzed at 488 nm in a BD *FACS Calibur* flow cytometer. All experiments were performed in triplicates.

4.3.7. Measurement of mitochondrial membrane potential (MMP) for compound 6a

MMP was measured by FACS analysis using abcam ab113852TMRE Mitochondrial Membrane Potential Assay Kit with DiOC2(3) staining using MitoProbe™ DiOC2(3) Assay Kit for Flow Cytometry (M34150). After treatment with compound **6a** at its IC_{50} concentration, cells were stained with DiOC2(3) dye for 30 min in the incubator, then harvested and washed with PBS. DiOC2(3)-stained cells were represented with Annexin binding buffer and incubated at 37 ^{o}C for 15 min. The samples were immediately analyzed using by cell Quest software.
